# Assessing medical student empathy in a family medicine clinical test: validity of the CARE measure

**DOI:** 10.3402/meo.v20.27346

**Published:** 2015-07-07

**Authors:** Julie Y. Chen, Weng Y. Chin, Colman S. C. Fung, Carlos K. H. Wong, Joyce P. Y. Tsang

**Affiliations:** 1 Department of Family Medicine and Primary Care, The University of Hong Kong, Pokfulam, Hong Kong; 2 Institute of Medical and Health Sciences Education, The University of Hong Kong, Pokfulam, Hong Kong

**Keywords:** empathy, validation, psychometric, undergraduate, medical student, primary care, assessment, clinical consultation, communication

## Abstract

**Introduction:**

The Consultation and Relational Empathy (CARE) measure developed and validated in primary care settings and used for general practitioner appraisal is a 10-item instrument used by patients to assess doctors’ empathy. The aim of this study is to investigate the validity of the CARE measure in assessing medical students’ empathy during a formative family medicine clinical test.

**Method:**

All 158 final-year medical students were assessed by trained simulated patients (SPs) – who completed the CARE measure, the Jefferson Scale of Patient Perceptions of Physician Empathy (JSPPPE), and a global rating score to assess students’ empathy and history-taking ability.

**Results:**

Exploratory and confirmatory factor analysis identified a unidimensional structure. The CARE measure strongly correlated with both convergent measures: global rating (*ρ*=0.79 and <0.001) and JSPPPE (*ρ*=0.77 and <0.001) and weakly correlated with the divergent measure: history-taking score (*ρ*=0.28 and <0.001). Internal consistency was excellent (Cronbach’s α=0.94).

**Conclusion:**

The CARE measure had strong construct and internal reliability in a formative, undergraduate family medicine examination. Its role in higher stakes examinations and other educational settings should be explored.

At the heart of a meaningful doctor–patient relationship is empathy (1). More than an expression of sympathy or a character trait, empathy in a clinical setting is a multifaceted concept. It includes emotive, moral, cognitive, and behavioral components (2) that can be articulated as a professional skill or competency – which in turn, can be learned, demonstrated, and assessed. It has a direct, positive impact on the quality of patient care (3) in terms of patient and doctor satisfaction, patient enablement, and possibly health outcomes (4).

Given its recognized importance in patient care, nurturing empathy from the earliest stages of medical training has been widely advocated, and the Association of American Medical Colleges has recommended that empathy be an essential objective in undergraduate education (5). One of the key aims of the undergraduate medical curriculum at the University of Hong Kong (HKU) is to develop students who will be able to ‘engage in productive, empathic relationships with patients, and display effective communication skills’ (6). Indeed, researchers have found that medical student empathy predicts future doctor–patient empathy, underlining the importance of cultivating the development of empathy in medical students during their training (7).

In terms of expected competencies within family medicine, medical students in their final year of undergraduate medical education at HKU are expected to be able to properly conduct a primary care consultation. This includes acquiring relevant information, generating diagnoses, and negotiating a management plan – all using a humanistic, patient-centered approach. Empathy is a central element in the patient-centered approach and key to the development of a therapeutic doctor–patient relationship. Since it is ultimately the patient’s perception which determines the success and effectiveness of the clinical relationship, patients’ perception of empathy is highly relevant.

On this premise, a patient-centered measure of empathy tailored to a primary care setting was developed in the UK. Known as the Consultation and Relational Empathy (CARE) measure, this 10-item questionnaire was designed to capture the set of physician competencies perceived by patients as important in holistic and empathic care (8). It has been subsequently validated in primary care settings in the both the UK (9) and Hong Kong (10) and is capable of distinguishing between doctors’ interpersonal competencies (11). In the UK, it also plays a role in quality assurance and training, where it is used for workplace appraisal and training of general practitioners (12).

Other measures of empathy, mostly general self-report instruments (e.g., the Interpersonal Reactivity Index (13), the Empathy Scale (14), the Emotional Empathy Scale (15)), have been used in a research context. The Jefferson Scale of Patient Perceptions of Physician Empathy (JSPPPE) (16) is a generic scale, which has been used in medical education – but is not specifically designed for primary care. As our focus is on clinical consultations conducted within a family medicine framework, the CARE measure is a more fit-for-purpose instrument which, if valid, may be a useful assessment tool in identifying deficiencies in medical students’ relational empathy, as perceived by their future patients.

The aim of this study, then, is to establish the validity of the CARE measure in assessing the empathy of final-year medical students during a formative family medicine clinical competency assessment.

## Method

### Subjects and setting

All final year medical students taking the formative family medicine clinical competency test (CCT) in 2013 comprised the target population. Administered at the end of each of six annual family medicine rotations, the CCT requires students to conduct a 15-min consultation with a simulated patient (SP) in the presence of an examiner. Every SP is trained to assess students on their interpersonal skills and empathy, and to assess students’ acquisition of key history-taking information using a case-based checklist.

SP training sessions were conducted prior to each CCT, and the content of the CARE measure was reviewed to ensure SPs understood each element they were required to assess. The SPs were encouraged to respond according to how the student actually made them feel during the consultation.

A total of nine SPs (three males and six females) assessed 8–10 students across 1–4 clinical rotations; all SPs were 20–30 years of age and of Chinese descent. Different SPs were used depending on the gender requirement for the case and/or SP availability.

All cases were structured similarly and based on a common complaint encountered in family practice (e.g., cough, headache, and palpitations) – requiring students to identify and address (in a management plan) a biopsychosocial problem list. Although some scenarios were more conducive to showing empathy, elements of the CARE measure (e.g., Does the student make you feel at ease? Does the student really listen to you? Does the student explain things clearly?) pertained to general interpersonal skills required of any consultation.

Written informed consent was obtained from students prior to the CCT, permitting the use of their assessment scores in the study.

### Study instrument

The CARE measure is a 10-item consultation process measure shown to produce valid scores of patients’ perceptions of relational empathy in primary care contexts (9). A 5-point Likert scale ranging from 1 (poor) to 5 (excellent) is used to rate each item, which are summed into a total score ranging from 10 to 50. Missing values were handled as recommended in the guidance notes on the scoring (12). Two or fewer missing values and ‘not applicable’ responses were replaced with the average score for the remaining items in that individual’s questionnaire. Questionnaires with more than two missing responses were excluded from the analysis.

### Comparison instruments

The *global rating of empathy* is a single question, which asked patients to give their overall impression of the student’s empathy, interpersonal connection, and attitude on a 5-point Likert scale. This item is based on a global rating scale for empathy, which has been used to assess physician empathy in the domains of patient connectedness – allowing patients’ sharing of feelings and perspective and showing of empathic expression (17). A similar summated global rating of senior medical student performance in the domains of empathy, coherence, and verbal/non-verbal expression has been shown to have good psychometric properties in an objective structured clinical examination (OSCE) setting (18).

The JSPPPE is a 5-item scale rated on a 7-point Likert scale describing empathetic engagement of the physician as perceived by patients. Its use in medical education has been supported by psychometric evidence in studies involving post-graduate medical trainees (16). It significantly correlates with patients’ satisfaction, interpersonal trust, and adherence to physicians’ recommendations (19) – and has also been used in a US medical school to assess empathy during a third-year OSCE (20).

A 10-item *history-taking checklist* documented student’s elicitation of key clinically relevant information from the SP. These items reflect solely factual information and are unrelated to interpersonal skills or empathy. Checklists completed by SPs or other observers have been useful in assessing history-taking and other domains in the realm of general medical practice (21).

### Ethics approval

Ethical approval of this study was granted by the Institutional Review Board of the University of Hong Kong/Hospital Authority Hong Kong West Cluster (Reference No.: UW 12-102).

### Data analysis

To identify potential floor or ceiling effects in the CARE measure, the proportions of students receiving the minimum and maximum possible scores were calculated to see if either exceeded 15% (22).

Using Spearman rank order correlation coefficients, construct validity of the CARE measure was established via its relationship to: 1) the JSPPPE and global empathy rating (convergent validity) and 2) the history-taking checklist (divergent validity). Convergent validity was supported if the CARE measure, the global empathy rating, and the JSPPPE scores were moderately to highly correlated (*r*≥0.3). Divergent validity was supported if the CARE measure was only correlated weakly (*r*<0.3) with the history-taking checklist score.

Exploratory factory analysis (EFA) utilizing a principal components method with Varimax rotation was used to establish the underlying factor structure of the CARE measure, and to compute the factor Eigenvalues and individual factor loadings. Factor loadings ≥0.5 reflected items’ correlation with a factor, while items which loaded <0.5 or loaded on multiple factors (i.e., cross-loaded) were removed from further investigation. Eigenvalues describe the amount of variance attributable to each factor; factors with eigenvalues of >1 were retained in the structure (23).

Confirmatory factor analysis (CFA) was performed to further examine the construct validity of the factor structure proposed by the EFA and the one-factor solution of the original (UK) version of CARE measure (9). Polychoric correlations measured the ordinal association between item scores, and maximum likelihood estimation explored the factor loadings and variance explained by one-factor solution. A chi-square test (24), goodness-of-fit index (GFI) (25), adjusted goodness-of-fit index (AGFI) (25), root mean square error of approximation (RMSEA) (26), and comparative fit index (CFI) were used to assess the model goodness-of-fit, which was considered adequate if: 1) chi-square test (*p*≥0.05); 2) RMSEA≤0.08; 3) GFI≥0.90; 4) AGFI≥0.80; and 5) CFI≥0.95 (27).

For factor analysis, the sample was split into two subsamples comprising only of cases with complete data (no missing responses). Data from rotations 1–3 and 4–6 were used EFA and CFA (respectively) to identify subscales. Cronbach’s α coefficient was used to determine each subscales’ internal consistency relative to the expected standard of ≥0.7 (28). The effect of imputed data substitutions (missing values) on internal consistency was undertaken in a sensitivity analysis.

Both the EFA and CFA were performed using LISREL 8.80 (Scientific Software International, Inc., Lincolnwood, IL, USA), while other statistical analyses were performed using IBM SPSS Window 21.0 program (SPSS, Inc., Chicago, IL, USA).

## Results

Of the 158 study subjects, 97 (61.4%) were male and ranged in age from 22 to 37 (median=24). Based on the six rotations of 2013 CCT examinations, the mean CARE measure score was 35.8 out of a possible 50. No floor or ceiling effects were observed. Descriptive, univariate statistics of key variables are shown in Table 1.

**Table 1:** Descriptive statistics of key variables

	Descriptive statistics		
	
	Mean	SD	Range
**CARE measure** total score (max 50)	35.8	7.3	17–50
1. Making patient feel at ease	3.6	0.9	2–5
2. Letting patient tell their ‘story’	3.6	0.7	2–5
3. Really listening	3.6	0.8	1–5
4. Being interested in patient as whole person	3.5	0.8	2–5
5. Fully understanding patient’s concerns	3.5	0.9	1–5
6. Showing care and compassion	3.6	0.8	1–5
7. Being positive	3.6	0.8	2–5
8. Explaining things clearly	3.6	1.0	1–5
9. Helping patient to take control	3.6	1.0	0–5
10. Making a plan of action with patient	3.5	1.1	0–5
**Patient’s global rating of empathy** (max 5)	3.6	0.7	2–5
**JSPPPE** total score (max 35)	23.7	5.0	9–34
1. Emotions	4.9	1.0	2–7
2. Concerned	4.4	1.2	1–7
3. Perspective	4.7	1.1	2–7
4. Daily life	4.8	1.2	1–7
5. Understanding	4.9	1.1	1–7
**History-taking checklist** total score (max 10)	7.9	1.4	2–10
1. Chief complaint	1.0	0.0	1–1
2. Started	1.0	0.1	0–1
3. Severity	0.9	0.3	0–1
4. Better	0.8	0.4	0–1
5. Worsen	0.8	0.4	0–1
6. Hospitalization	0.8	0.4	0–1
7. Medication	0.8	0.4	0–1
8. Marital status	0.4	0.5	0–1
9. Smoke	0.7	0.5	0–1
10. Diagnosis	0.8	0.4	0–1

### Exploratory and confirmatory factor analysis

The validity of our data was first confirmed using EFA where the Kaiser–Meyer–Olkin measure of 0.94 and Bartlett’s test of sphericity [*χ* ^2^(45)=887.8, *p*<0.001] confirmed the sampling adequacy and variability. Using a principal components analysis, a one-factor solution was shown to explain 77.6% of the total variance. All 10 items loaded significantly on this single factor.

Based on conventional guidelines, a CFA found that this one-factor model met the criteria demonstrating excellent goodness of fit (RMSEA=0.06; GFI=0.89; AGFI=0.83; CFI=0.99). The null hypothesis of chi-square test was rejected (*χ* ^2^=46.72; *p*=0.09), suggesting an adequate fit of the data with the one-factor model. EFA and CFA loading are shown in Table 2.

**Table 2:** Exploratory and confirmatory factor loadings of CARE measure items

Items	Exploratory factor analysis (*n*=79) Factor loading^a^	Confirmatory factor analysis (*n*=73)	

		Factor loading^a^	Variance explained
1. Making patient feel at ease	0.915	0.991	0.983
2. Letting patient tell their ‘story’	0.894	0.995	0.991
3. Really listening	0.908	0.990	0.981
4. Being interested in patient as whole person	0.873	0.992	0.983
5. Fully understanding patient’s concerns	0.911	0.981	0.963
6. Showing care and compassion	0.929	0.993	0.985
7. Being positive	0.881	0.990	0.98
8. Explaining things clearly	0.821	0.986	0.973
9. Helping patient to take control	0.777	0.990	0.979
10. Making a plan of action with patient	0.868	0.980	0.961
Eigenvalue^b^	7.722		
% of variance	77.224		

a Factor loading of ≥0.5 was used as the cut-off to sort items into factors.

b Eigenvalue ≥1 was used to determine the number of factors. *Goodness-of-fit indices*: root mean square error of approximation (RMSEA)=0.0589; goodness-of-fit index (GFI)=0.892; adjusted goodness-of-fit index (AGFI)=0.830; comparative fit index (CFI)=0.996; chi-square test=46.725; *p*=0.089. *Cut-offs used to indicate goodness of fit*: RMSEA ≤0.08; GFI≥0.9; AGFI≥0.8; CFI≥0.9.

### Convergent and divergent validity

Patients’ total CARE measure scores were strongly positively correlated with both their global empathy rating (*ρ*=0.79 and <0.001) and the JSPPPE scores (*ρ*=0.77 and <0.001), but only weakly associated with the history-taking score (*ρ*=0.28 and <0.001). This is shown in Table 3.

**Table 3:** Correlation of total CARE measure score with convergent and divergent constructs

	Spearman’s rho (*ρ*)^a^	*p*	*N*
Convergent constructs			
Patient’s global rating of empathy	0.794	<0.001	157
JSPPPE	0.771	<0.001	157
Divergent construct			
History-taking score	0.277	<0.001	158

JSPPPE=Jefferson Scale of Patient Perceptions of Physician Empathy.

a Cut-offs for Spearman’s rho (ρ): ≥0.70=very strong correlation; 0.40–0.69=strong correlation; 0.30–0.39=moderate correlation; 0.20–0.29=weak correlation.

### Internal consistency

Internal consistency of the 10-item CARE measure was excellent, as evidenced by the Cronbach’s α of 0.94. A sensitivity analysis of mean substitutions of missing data yielded only a miniscule increase in internal consistency (α=0.95).

## Discussion

The CARE measure is a widely used means of assessing primary care doctors’ relational empathy during a consultation, from the patient’s perspective. In this study, we extrapolated its validity to include medical students’ consultations in an undergraduate family medicine setting – showing that the CARE measure retained its original unidimensional structure (9), excellent internal consistency, and had good convergent and divergent validity. These findings bring the patient perspective squarely into medical educational assessment and should encourage more objective and standardized assessment of a complex attribute, empathy, in a formative (low-stake), family medicine context.

As validated in this context, the CARE measure may have some educational benefits over shorter measures like the 1-item global empathy rating or the 5-item JSPPPE. Firstly, with 10 items, the CARE measure expands a complex concept into a set of concrete, practical elements that are clearly understood by students. Smaller components enable students to focus on particular aspects of the whole, analogous to learning a complex skill through microskill acquisition. Secondly, items better articulate the interpersonal skills needed by primary care doctors, so its applicability in primary care would be an advantage in teaching consultations in family medicine and other primarycare-oriented settings. Similar to some instruments used to measure healthcare outcomes – shorter, generic measures may not have the sensitivity to capture small differences or may be less responsive to capturing changes over time in a specified population (29). Used formatively, where the focus is to help students learn and improve, the CARE measure can serve as a guiding rubric that represents the essential elements desired in a primary care consultation. This may be used for benchmarking and for generating student feedback to help identify specific clinical strengths and weaknesses.

Furthermore, the absence of a floor or ceiling effect in this context may make this instrument sensitive enough to differentiate among students’ performance. In contrast, when used in doctor–patient or therapist–patient settings, CARE measure scores tended toward the higher end of the distribution – with more than a quarter of targets receiving the maximum score (9, 30). Real patients are likely to voluntarily seek out and establish relationships with doctors they find ‘acceptable’ and whom they may already know well. For students, this is a required interaction that represents a one-off visit. As well, SPs in an undergraduate exam setting may recognize the ‘developmental’ limitations of students, and hence refrain from awarding them the maximum score.

The excellent internal consistency of the CARE measure found in this study provides some preliminary evidence for its reliability. However, assessments of the same student by multiple examiners or over time would offer additional support of its reliability. In the primary care setting, it has been suggested that 50 completed assessments by patients using the CARE measure are required to reliably assess doctors’ empathy (8), which would be impossible or impractical in most educational settings.

The value of assessment for learning (as opposed to assessment of learning) has been advocated in the learning of clinical competencies in medical education (31), and students’ relational empathy may be best developed and improved if assessed in the same way. The CARE measure provides a valid way in which students can be assessed and learn to improve their relational empathy. This, combined with qualitative feedback from peers/supervisors and self- reflection, can provide a more solid indication of students’ acquisition of a core clinical consultation skill.

### Strengths and limitations

An adequate and appropriate sample, as well as the use of external measures to establish convergent and divergent validity, is among the strengths of this study.

The main limitation relates to the unknown generalizability of the findings to other educational settings or activities. Even though our study included a low-stakes, formative emphasis, both students and patients may have behaved differently than those within a more realistic clinical setting. In addition, our study was conducted in a specific setting, in one curriculum, and at one institution, which necessitates further study to examine validity issues in other educational settings. Finally, although the internal consistency of the CARE measure was established, further psychometric examination in terms of test–retest and inter-rater reliability would greatly strengthen our findings.

## Conclusion

The CARE measure was shown to have strong construct validity and excellent internal consistency in a formative, undergraduate family medicine examination. It also has some discriminatory potential in this context due to the absence of floor or ceiling effects and the ability of SPs to complete the measure under exam conditions. This study demonstrated that the CARE measure can be a useful tool to assess and generate feedback to students on specific interpersonal elements of the consultation – bringing patients’ perspective into the realm of primary care consultation. Further work is needed to explore its role in higher stakes clinical examinations and other educational settings.
